# Glaucoma and Alzheimer: Neurodegenerative disorders show an adrenergic dysbalance

**DOI:** 10.1371/journal.pone.0272811

**Published:** 2022-10-06

**Authors:** Bettina Hohberger, Harald Prüss, Christian Mardin, Robert Lämmer, Johannes Müller, Gerd Wallukat

**Affiliations:** 1 Department of Ophthalmology, Universität of Erlangen-Nürnberg, Friedrich-Alexander-University of Erlangen-Nürnberg, Erlangen, Germany; 2 Department of Neurology, Charite´-Universitätsmedizin Berlin, Berlin, Germany; 3 Berlin Cures GmbH, Berlin, Germany; Torrey Pines Institute for Molecular Studies, UNITED STATES

## Abstract

Glaucoma disease is characterized by an increased intraocular pressure (IOP), glaucomatous alterations of the optic disc and corresponding visual field defects. Even lowering the main risk factor IOP until an individual target level does not prevent this neurodegenerative disorder from proceeding. Several autoimmune mechanisms were discovered, partly showing a functionality. One of these autoimmune phenomena targets the ß2-adrenergic receptor (ß2-AR; i.e. agonistic autoantibodies; ß2-agAAb) and is linked to an elevated IOP and an impaired retinal microcirculation. As neurodegenerative disorder, Alzheimer’s Disease (AD) is postulated to share a common molecular mechanism with glaucoma. In the present study we investigated autoimmune phenomena targeting the ß2-AR in patients with AD. Sera of the patients were analyzed in a rat cardiomyocyte bioassay for the presence of functional autoantibodies against ß2-AR. In addition, different species of amyloid beta (Aß) monomers were tested (Aß1-14, Aß10-25, Aβ10–37 Aß1-40, Aß1-42, Aβ28–40, and Aß-[Pyr]3–43). Our results demonstrate that none of the short-chain Aß (Aß1-14, Aß10-25, or Aβ28–40) showed any agonistic or inhibitory effect on ß2-AR. Contrary, long-chain Aß-[Pyr]3–43, representing a major neurogenic plaque component, exerted an activation that after blocking by the ß2-AR antagonist ICI118.551, could be identified as that the effect was realized via the ß2-AR. Moreover, the long chain Aß1-40, Aβ1–42, and Aβ10–37, yet not the short-chain Aß peptides prevented the clenbuterol induced desensitization of the ß2-AR. In addition, we identified functional autoantibodies in the sera of AD patients, activating the ß2-AR, like the ß2-agAAb found in patients with glaucoma. As autoimmune mechanisms were reportedly involved in the pathogenesis of glaucoma and Alzheimer’s Disease, we postulate that overstimulation of the ß2-AR pathway can induce an adrenergic overdrive, that may play an important role in the multifactorial interplay of neurodegenerative disorders.

## Introduction

Glaucoma disease is a worldwide burden, being the second leading cause of blindness in the industrial nations. This neurodegenerative disease is characterized by an increased intraocular pressure (IOP), a glaucomatous optic disc and visual field defects, going along with disease progression. Up to now the exact pathomechanisms are still elusive. An elevated IOP, the main risk factor, is the target of conservative, laser and surgical therapy. Yet, despite lowering IOP, glaucoma disease proceeds, as no causal therapy is available until now [1]. Neurodegeneration in glaucoma is featured by apoptosis of the retinal ganglion cells (RGC). Immune and autoimmune mechanisms can interplay in these molecular interactions of loss of RGC as recent studies suggested. Data of mice models yielded a significant RGC loss mediated by T-lymphocytes via heat-shock-proteins in the presence of only slightly elevated IOP [2]. Changed cytokine levels [3] and microglial activation were observed in a mice model of ocular hypertension [4]. Even normotensive eyes were observed to show an apoptosis of RGC when the contralateral eye was hypertensive (mice model). This effect was attributed to lymphocytes as well [5]). Apoptosis itself, a vascular dysregulation or other extern factors (e.g. nutrition, alteration of levels of trace elements) are able to trigger immune and autoimmune mechanisms. Thus, it is not surprising that diverse autoantibodies were detected in sera of patients with glaucoma. In this context, autoantibodies with a functional consequence are of special interest. Recent studies presented autoantibodies targeting the adrenergic ß2-receptor (ß2-AR) in sera of patients with open-angle glaucoma. These ß2-autoantibodies activate their target (ß2-agAAb) with consecutive overstimulation and desensitization of the receptor contrary to physiological agents. *In vivo* data suggested a link of the ß2-agAAb to the increased IOP and impaired retinal microcirculation [6,7]. We propose that a chronic overstimulation of the ß2-AR can result in a dysregulated homeostatic adrenergic balance. Receptor desensitization and internalization might be only one of the molecular features [8]. If this adrenergic disbalance has sufficient ‘power’ on cellular level, clinical characteristics occur in the patients (*imbalanced autonomic theory*; see also [9]). This adrenergic disbalance is certainly not only a local problem (in the eye). We assume that adrenergic alterations might be even in extraocular tissue of patients with neurodegenerative diseases.

Patients with Alzheimer’s Disease (AD) show amyloid beta (Aß) depositions being proposed to be the major mediator of neurotoxicity in the brain [10]. Aß is assumed to be one crosslink between glaucoma and AD. RGC express Aß [11,12]. Especially, intravitreal injection of Aß1-42 was observed to induce RGC loss [13]. Aß is generated after cleavage of the amyloid precursor protein (APP). Ni et al. has shown that a ß2-adrenergic stimulation that activate the gamma secretase is involved in the formation of Aß from the APP [14]. This cleavage can be mediated by vessel endothelial cell enzymes and platelets, especially when platelets contact the endothelium. Any thrombosis can enhance this Aß generation. Any thrombosis can enhance this Aß generation.

Glaucoma and AD disease were observed to share common features clinically and biochemically. Patients with open-angle glaucoma are at higher risk [15]and showed a higher incidence of AD [16]. Vis-a-vis patients with AD are at higher risk of glaucoma [17]. Both, glaucoma and AD, were observed to be age-related disorders [18–22]. As the eye, especially retinal tissue, represents “cerebral tissue” considering embryology, it is not remarkable that neurodegeneration and amyloid-β plaques were seen within the retina in AD patients [23–25]. Since several years a common pathomechanism of glaucoma and AD has been suggested [26–28]. Patients with AD showed amyloid beta (Aß) depositions being proposed to be the major mediator of neurotoxicity in the brain [10]. Aß is assumed to be one crosslink between glaucoma and AD. RGC express Aß [11,12]. Especially, intravitreal injection of Aß-42 was observed to induce RGC loss [13]. Degeneration of RG and retrograde degeneration of axons in the lateral geniculate body are two findings, being present in AD patients [13,25,29,30]. Even amyloid-ß can induce RGC loss within the retina itself [13]. These neurodegenerative features were also observed in glaucomatous eyes [31–34]. The diagnostic correlate of RGC loss can be seen in retinal nerve fiber layer thinning (RNFL) *in vivo*, visualized by magnet resonance tomography and optical coherence tomography (OCT) [31,33]. Both, patients with glaucoma and AD, showed a RNFL thinning especially in the superior and/or inferior retina [35–38]. Thus, it can be hypothesized that there is common pathway, linking both neurodegenerative disorders (glaucoma and AD). Functional AAb against the ß2-AR were not only seen in the sera of patients with glaucoma but also in sera of patients with AD symptoms [39]. These ß2-agAAb but also the long chain Aß peptides prevent the desensitization of the ß2AR and induce a permanent stimulation of this adrenergic receptor. As ß2-agAAb show a link to an impaired microcirculation (glaucoma) and Aß expression is increased during impaired microcirculation (i.e. during and after thrombosis; AD), we hypothesize that there is a common molecular mechanisms between adrenergic dysfunction (ß2-agAAb) and Aß. The aim of the study was to investigate sera of patients with AD and glaucoma for the presence of autoantibodies against ß2-AR, and the effect of fragments and the complete Aß peptides on the ß2-AR and whose desensitization.

## Material and methods

### Patients and controls

Sera of 11 subjects with AD (3 female, 8 male, age: 40–87 years) and 18 subjects with primary open-angle glaucoma (7 female, 11 male, age: 42–76 years) were collected for the present study. Glaucoma patients were recruited at the Department of Ophthalmology, University of Erlangen-Nürnberg, Friedrich-Alexander-University Erlangen-Nürnberg [Erlangen Glaucoma Registry, ISSN 2191-5008, CS-2011; NTC00494923] [1]. Patients with AD were recruited at the Department of Neurology, Charite´-Universitätsmedizin Berlin. Blood samples were collected and centrifuged, and sera were stored at -20°C. All individuals declared informed consent. The study was performed according to the tenets of the Declaration of Helsinki and approved by the ethic committee of the university of Erlangen, the institutional Review Board of Charite´–Universitätsmedizin Berlin and the Max-Delbrück-Centrum for Molecular Medicine, Berlin-Buch (Y 9004/19, 02.11.2020).

### Primary Open-Angle Glaucoma (POAG) patients

The criteria of POAG were: an open anterior chamber angle, IOP>21 mmHg (confirmed at least once, measured by Goldmann applanation tonometry), and a glaucomatous optic disc, classified after Jonas [40]. Functional loss of perimetric field was measured and had to be confirmed at least once according to the following criteria: (I) at least three adjacent test points having a deviation ≥5 dB and with one test point with a deviation >10 dB lower than normal, or (II) at least two adjacent test points with a deviation ≥10 dB, or (III) at least three adjacent test points with a deviation ≥5 dB abutting the nasal horizontal meridian or (IV) a mean visual field defect of >2.6 dB.

### Patients with AD

In the study we included 11 patients (3 female,8 male) with diagnosed AD with a mean age of 70 years. All the investigated patients had hypertension and 3/11 patients had coronary heart disease. One patient was diseased with a peripheral artery disease, 7/11 patients were treated with antidementia drugs.

### Cardiomyocyte bioassay

The cardiomyocyte bioassay was done as described previously (Junemann et al., 2018). Cell culture of cardiac myocytes of heart ventricle (1–3 day-old Sprague-Dawley rats) was used. digested with a 0.25% solution of crude porcine trypsin (Serva, Germany). The cells were dispersed by trypsinization and suspended in a SM20-I medium (Biochrom, Germany), glutamine (Serva, Germany), containing streptomycin (HEFA Pharma; Germany), penicillin (Heyl, Germany), hydrocortisone (Merck, Germany), fluorodeoxyuridine (Serva, Germany), and 10% heat-inactivated neonatal calf serum (Gibco, Germany). The cells were seeded (field density of 160.000 cells/cm^2^) the culture medium was replaced after 24 hours. The cardiomyocytes were cultured for 3–4 days (37°C) before being used in the experiments. Firstly, the basal beating rate of the cardiomyocytes was measured for 15 seconds; at a heated stage of an inverted microscope at 37°C visually or with an ION OPTIX measurement place. Subsequently the immunoglobulin (Ig) fractions were added for 60 min in a dilution of 1:40. Next, the beating rates (BR) of the cardiomyocytes were counted again on 6 marked spontaneously beating cells or cell cluster. The data are expressed as “increase in number of beats/15sec”. The calculated cut off value was 1.83. Application of a specific β2-blocker, i.e. ICI 118.551 (0.1μM) was done for identification of the receptor type.

### Preparation of the serum immunoglobulins

Human IgG were prepared from each patients’ sera by direct ammonium sulfate precipitation (final concentration of 40%; overnight at 4°C). The precipitates were centrifuged (4,000 × g; 30 min) and the pellets were dissolved in dialysis buffer (154 mmol/l NaCl, 10 mmol/l Na_2_HPO_4_/NaH_2_PO_4_, pH 7.2). This procedure was repeated twice. Each sample was dialyzed against phosphate-buffered saline (4°C; 3 days). Storage of the samples was done at −20°C.

### Desensitization experiments

In these experiments the cardiomyocytes were treated with1μM clenbuterol with and without different Aβ-peptides and with AAb directed against the β2-adrenoceptor. The incubation time was 120 min followed by a washing procedure and anew stimulation with clenbuterol at the same concentration. In this experiment we used AAb prepared from the sera of AD patients and the short- and long-chain Aβ-peptides: Aβ1–14, Aβ25–35, Aβ28–40, Aβ10–37, Aβ1–40, and Aβ-1-42.

### Aß peptides

The fragmented Aß peptides were synthesize by Dr. Beyermann (FMP Berlin). The long-chain Aß peptides Aß 1–40, Aß 1–42 and also Aß-[Pyr[3–43 were from Sigma-Aldrich.

### Statistical analysis

Statistical analysis was done by SPSS (version 21) and Excel and the unpaired t-test. Data are shown as absolute value, mean and standard deviation.

## Results

### β2-AAb in patients with glaucoma and Alzheimer’s disease

**Fig 1:**
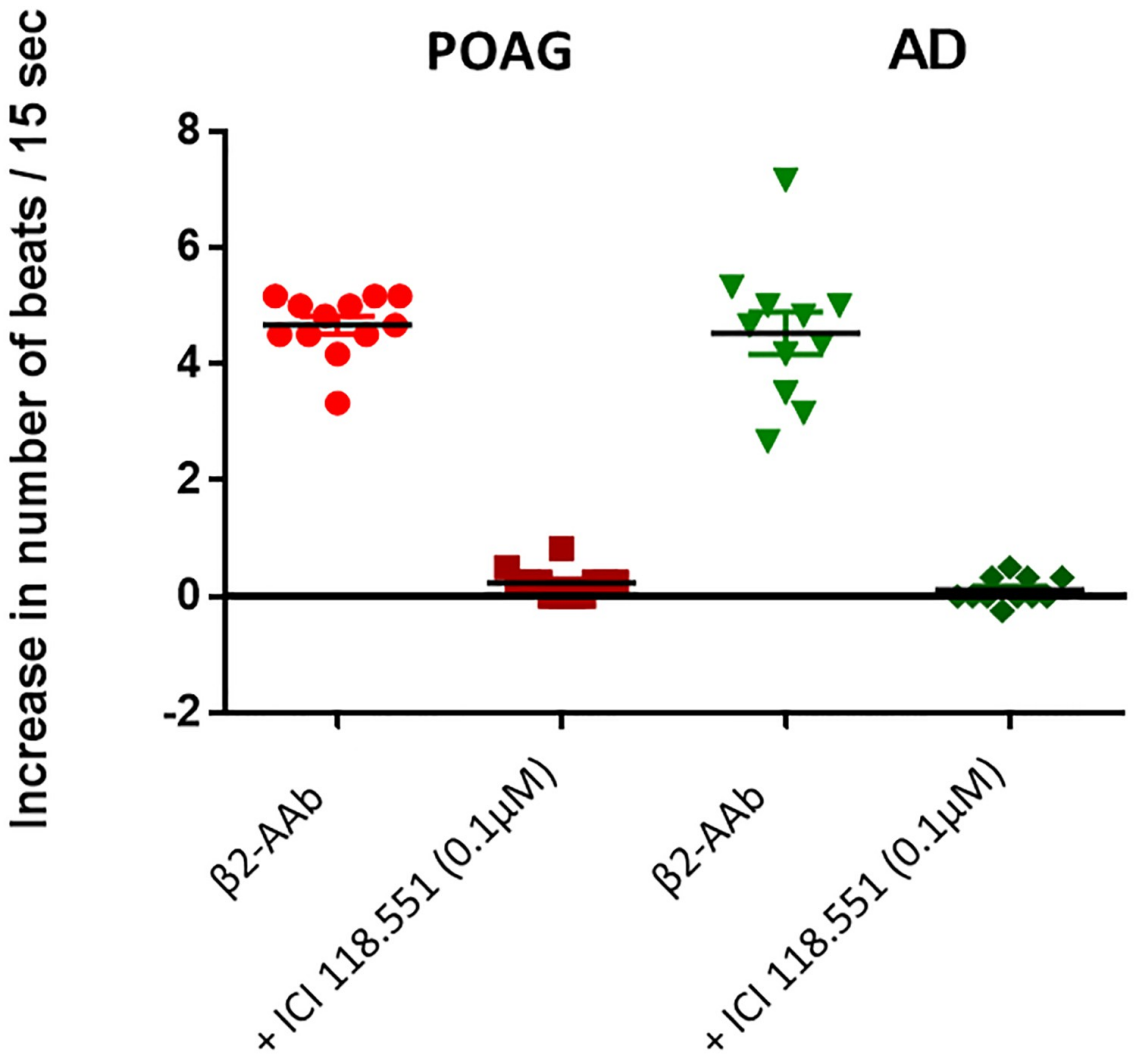
Comparison of the activity of the functional β2-adrenoceptor autoantibodies (β2-AAb) of patients with glaucoma (POAG) (n = 12) and Alzheimer’s disease (AD) (n = 11). Both agonist-like effects were blocked by the β2-adrenoceptor antagonist ICI 118.551 (0.1μM), (p˂ 0.001). The experiments with the AAb of the AD patients were done in the presence of the α1- adrenoceptor antagonist urapidil to block the agAAb against the α1-adrenoceptor that are also present in the sera of AD patients.

AAbs prepared from patients with POAG showed a mean beat rate of 4.5±0.1 U (range: 3.3–5.2 U) that was realized via the β2-AR. This increase in the beat rate could be blocked by the specific ß2-blocker ICI 118.551 (Fig 1). Immunoglobulins prepared from AD patient sera showed an increase of the mean beating rate of 4.5±0.4 U (range: 2.3–7.2 U). As observed for patients with POAG, this increase in the beat rate could be blocked by the specific ß2-blocker ICI 118.551 (Fig 1). These AAb recognize also the β2-adrenoceptor. Thus, a seropositivity for β2-AAb was observed for patients with POAG and AD. Healthy individuals, showing a seronegativity of G-Protein coupled receptors (GPCR)-AAb, have been tested in our previous works using the same assay [7,41].

### In vitro analysis of the effects of amyolid ß (Aβ) peptides

**Fig 2:**
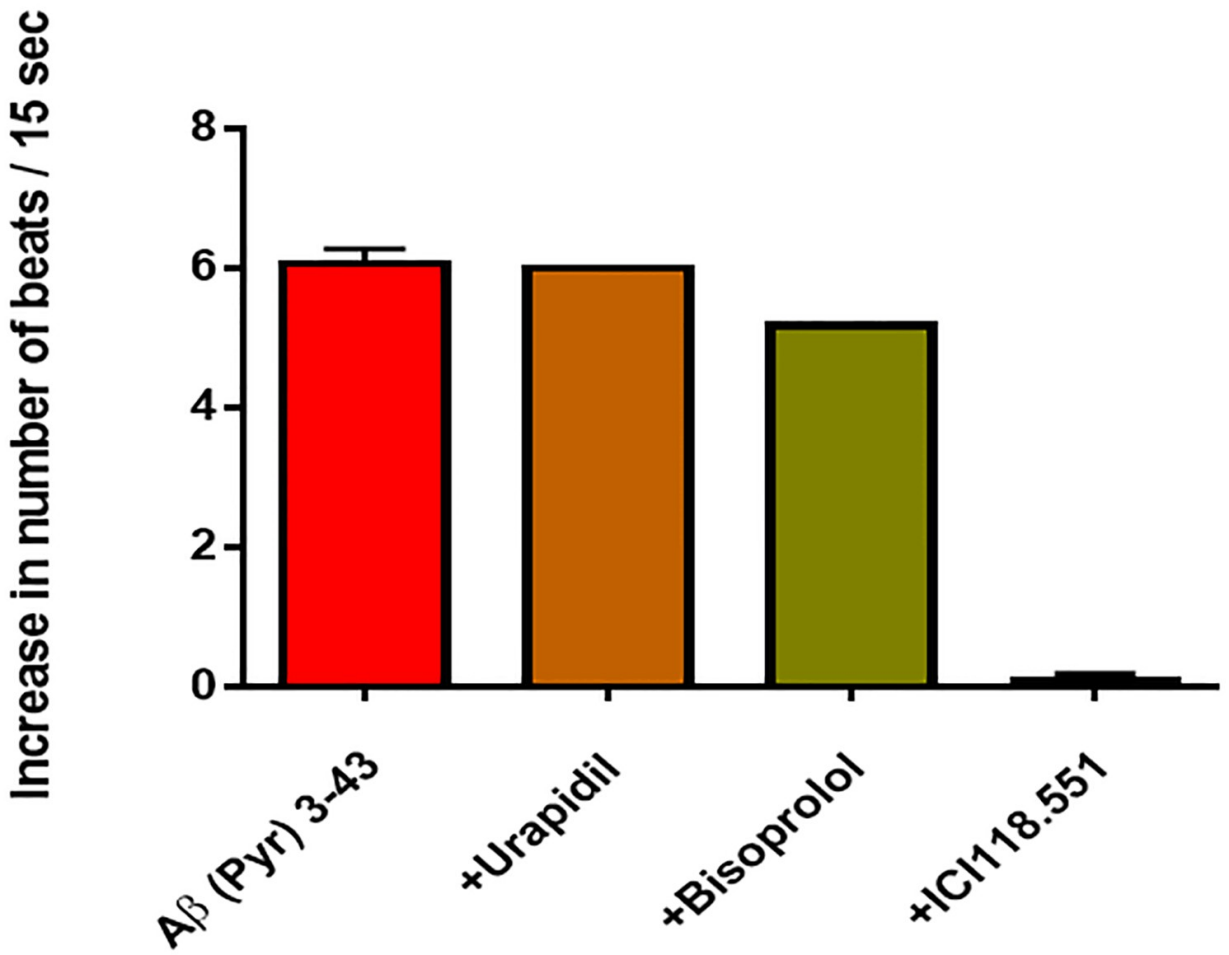
Functional effect realized via the β2 adrenoceptor by the truncated Aβ peptide Aβ-[PYR]3–43 (n = 5): This effect was blocked by ICI 118.551 (n-4, p˂0.001), yet not by the β1 adrenoceptor antagonist bisoprolol or the α1-adrenergic antagonist urapidil.

In order to identify a possible functional action of Aß peptides on the GPCR, different species of amyloid beta (Aß) peptide were tested in the used bioassay. Following Aß peptides were investigated in a concentration of 0.1μM: Aß1-14, Aß10-37, Aß 25–35, Aß 28–40, Aß1-40, Aß1-42, and Aß-[Pyr]3–43). Our results demonstrate that only the Aß peptide Aß-[Pyr]3–43, but not the other Aß peptides or Aß fragments are able to activate the β2 adrenoceptor similar as the classical β-adrenergic agonists. The long-chain Aß-[Pyr]3–43, representing a major neurogenic plaque component, exerted an activation of the ß2-AR blocked by the ß2-AR antagonist ICI 118.551 but not by the β1-AR antagonists bisoprolol or the α1- AR blocker urapidil (Fig 2).

### Desensitization experiments

**Fig 3:**
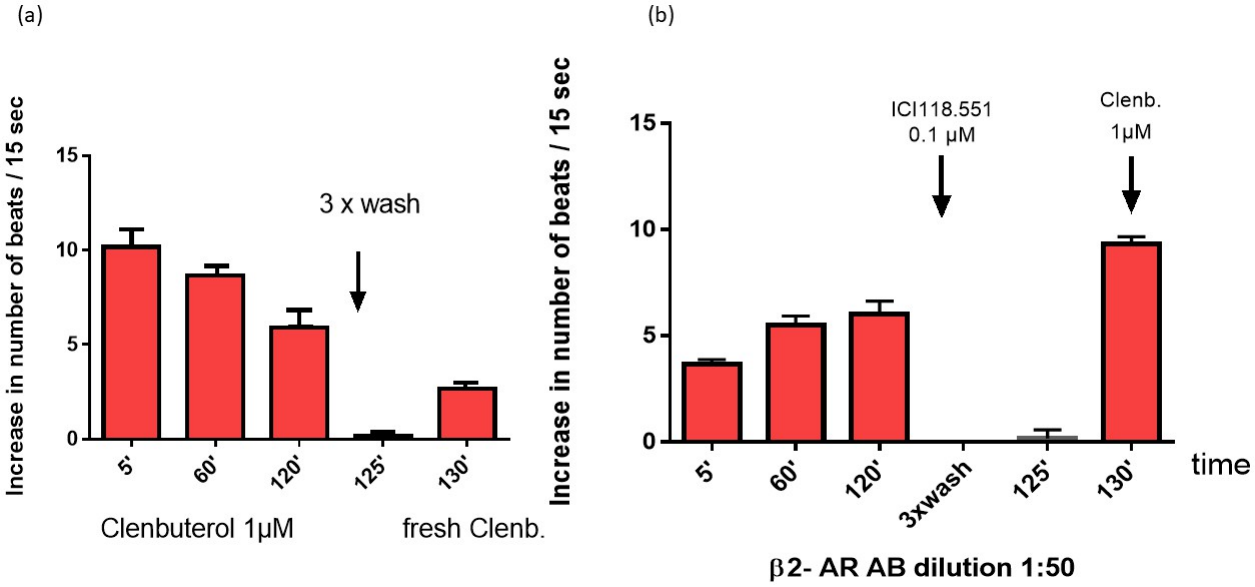
Desensitization of the β2 adrenergic response by the β2-adrenergic agonist clenbuterol (a). This receptor desensitization was missed if the cells were stimulated with the β2-adrenergic AAb prepared from AD patients (b).

**Fig 4:**
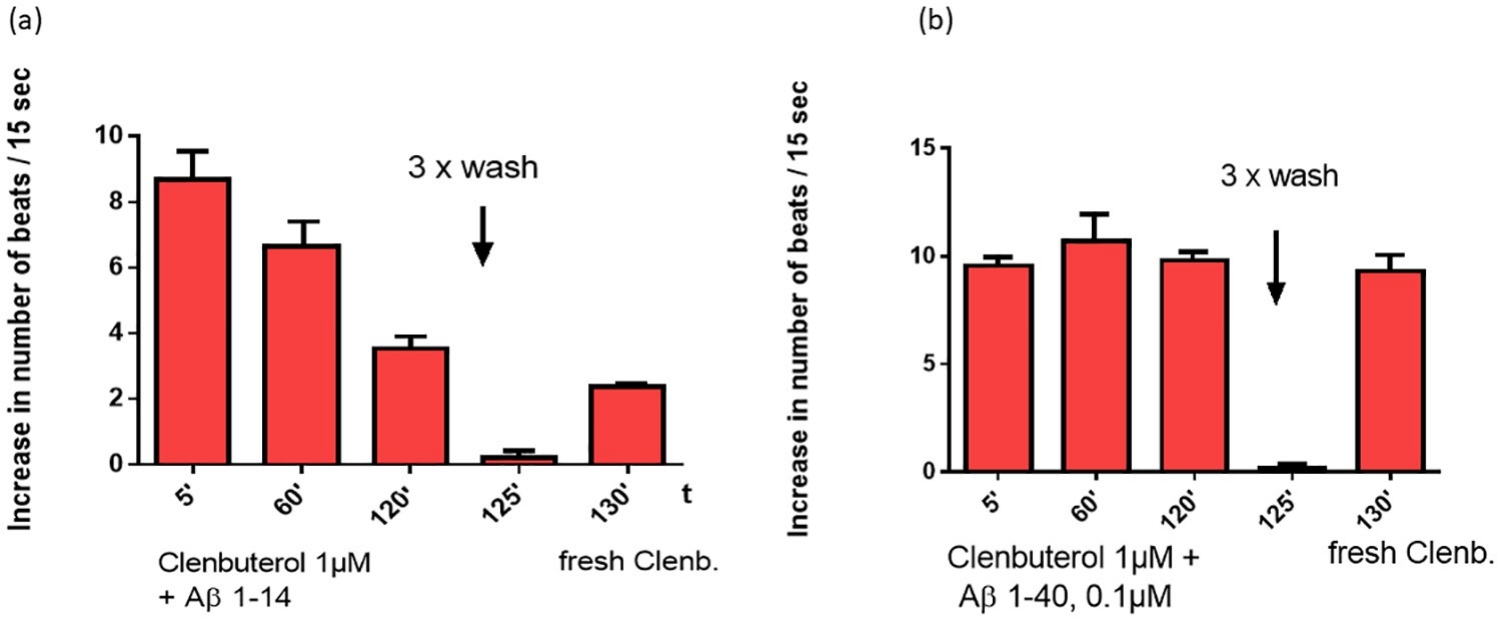
Influence of the Aβ peptides on the clenbuterol induced β2-adrenoceptor desensitization: The desensitization of the β-AR response by clenbuterol, was not influenced by the short chain Aβ peptides (a). However, the long chain Aβpeptides Aβ 10–37, Aβ1–40, and Aβ1–42 prevent the desensitization and exert a permanent stimulation of the β2-AR signal cascade (b).

A long-term incubation of the cardiomyocytes with adrenergic agonists leads to desensitization of the adrenoceptors and to a resistant to the used drug. The incubation of the cardiomyocytes with the β2-adrenergic agonist clenbuterol caused a time dependent reduction of the response to this agonist. A washing procedure removed this agonist completely and a new stimulation with 1μM clenbuterol generate only a strong reduced response (Fig 3a). Yet this protection mechanism of the cell can be disturbed by agAAb directed against these receptors and by long-chain amyloid peptides. Additionally, β2-agAAb caused a positive chronotropic effect that could not be washed out by a washing procedure. Therefore, the agAAb against the β2-adrenoceptor must be blocked and removed from the receptor by the specific β2-adrenoceptor antagonist ICI118.551. After the washing procedure the addition of clenbuterol led to a maximal stimulation. These data demonstrate that the β2-agAAb prevent the desensitization of the receptor (Fig 3b). Similar results were observed, if the long chain Aβ peptides plus clenbuterol were added to the cardiomyocytes. Under these conditions the β2-adrenoceptor was not desensitized and after the washing procedure the clenbuterol effect was not diminished (4b, Tab1). Contrary, in the presence of the short-chain Aβ peptides clenbuterol desensitized the β2 adrenoceptor like clenbuterol itself Fig 4a, Table 1).

**Table 1:** Influence of short and long chain Amyloid β peptides on the clenbuterol induced β2-adrenoceptor desensitization.

**Time**		**5’**	**60’**	**120’**	**3 x wash**	**125’**	**Clenbuterol 1 μM**	**130’**
**Clenbuterol 1 μM**	Clenbuterol 1μM	10.2±0.3	8.7±0.3	5.9±0.3		0.2±0.1		2.7±0.1
	+ Aβ 1–14	8.7±0.6	6.7±0.5	3.5±0.5		0.2±0.1		2.4±0.1
	+ Aβ 25–35	8.9±1.2	7.4±1.0	5.7±0.6		0.2±0.1		2.9±0.2
	+ Aβ 28–40	10.5±0.2	7.8±0.6	4.3±0.6		0.3±0.2		2.3±0.2
	+ Aβ 1–40	10.8±0.2	10.7±0.4	9.8±0.2		0.6±0.1		9.3±2.9
	+ Aβ 1–42	8.9±0.1	9.3±0.4	8.9±0.4		0.2±0.1		9.6±0.3
	+ Aβ 10–37	9.6±0.2	10.1±0.3	8.2±0.8		0.4±0.1		10.3±0.2

## Discussion

Glaucoma disease is one of the leading causes of irreversible blindness worldwide [42]. Its complex pathogenesis is known to be multifactorial with an immune and autoimmune involvement. Clinical studies observed that this neurodegenerative ocular disorder shows several common features with neurodegenerative disorders of the brain (e.g. AD)) [26,27,43,44]. Thus, it is commonly accepted that both disease entities seem to have common molecular pathways and clinical characteristics. To the best of our knowledge the present study offers data of a common pathogenic involvement of the β2-adrenergic pathway both in AD and glaucoma. In sera of patients with AD and glaucoma a seropositivity for ß2-agAAb was observed, respectively. In addition, the long-chain Aβ-[Pyr]3–42, representing the major neurogenic plaque component, showed a direct activation of the ß2-AR that was blocked by the specific antagonist ICI118.551. This N-terminal truncated isoform of Aβ can trigger neurodegeneration in a transgenic mouse model [45]) and developed a high neurotoxicity [46]. The other functional active Aβ peptides realize their effect in the used test model not via the β2 adrenoceptor. Another aspect of the effects of the Aβ peptides exist in fact that the long chain Aß1-40, Aβ1–42, Aβ10–37 peptides prevented the clenbuterol induced desensitization of the ß2-AR similar as the β2-adrenoceptor autoantibodies. All three mechanisms result finally in a boosted activation and permanent stimulation of the ß2-adrenergic pathways, potentially causing a dangerous adrenergic overdrive realized via the β2-AR.

The autonomic system is a main actor in human body. It regulates important function and is the basis of human life. Any alterations in the sympathetic and/or parasympathetic pathways results in an impairment or overaction of the autonomic system. Adrenergic receptors are also present on pericytes thus it can be hypothesized that any alterations in adrenergic activity might influence and alter microcirculation. It is commonly accepted that an impaired microcirculation can induced amyloid plaques in AD [47–49] and is a very early pathogenetic alteration in glaucoma disease [50–52]. Up to now, it is still elusive which factors induce this impaired microcirculation in AD. Patients with AD showed an early involvement of the choroidal microcirculation [53]. These clinical data were repeated in different murine models of AD (e.g. App NL-F) [54]. In addition, alterations in all retinal layers were observed, being dependent on whether patients have mild or moderate AD [55]. Interestingly, recent data showed that an impaired microcirculation is able to change the amyloid-β plaques in a mouse model [49]. Even in glaucoma pathogenesis, the exact pathogenesis is under investigation. There is evidence that an involvement of the autonomic system, especially the adrenergic ß2-mediated pathway might be present in dysregulation of IOP (glaucoma) [56] and capillary microcirculation (glaucoma [6] and AD), as patients with glaucoma and AD showed a seropositivity for ß2-AAb, respectively.

In patients with AD disease the ß2-adrenergic dysregulation might be mediated in three different ways. First is the direct activation of the ß2-AR by its classical natural agonist adrenaline or noradrenaline. Both compounds activate the ß1-, the ß2- and the ß3-AR. The stimulation with adrenaline, isoprenaline or the more ß2-AR specific agonist clenbuterol induced a positive chronotropic response in the cardiomyocyte bioassay, which could be blocked by the specific ß2-AR antagonist ICI118.551. It was shown that patients with AD develop functional active autoantibodies directed against the ß2-AR. These ß2-agAAb activate the ß2-AR in a similar manner as the ß2-adrenergic agonists [39], but in contrast to the agonists the ß2-agAAb prevent the desensitization of the corresponding receptor. This observation was also made in glaucoma patients [7]. The agonist like effect of this ß2-agaAAb was blocked by ICI118.551, yet not by the β1-AR antagonist bisoprolol. Moreover, the agonistic effect of the ß2-agAAb was neutralized by a peptide corresponding to the second extracellular loop of this receptor [7]. However, the β2-AR agAAB prepared from AD patient sera recognize the first extracellular loop of the β2-AR [39]. In contrast to the classical ß-AR agonists, ß2-agAAb activate the ß2-AR mediated signal cascade permanently. Moreover, the ß2-agAAb also prevent the desensitization, normally seen for the classical ß2-AR agonists, when agonists were added to ß2-agAAb pre-stimulated cardiomyocytes. This permanent stimulation without desensitization of the β2-AR may represent a pathogenic factor that played an essential role in the pathogenesis of several diseases [57]. Another feature of these ß2-agAAb is the possible antagonistic action of these ß2-agAAb directed against the β2-AR. It was shown by Mijares et al. that monovalent Fab fragments of monoclonal β2-agAbbs exhibit antagonistic and not agonistic properties [58]. However, if these monovalent Fab fragments were crosslinked by an anti Fab antibody than the agonist-like effects were restored [59]. These data demonstrate that the ß2-agAAb also can act in an antagonistic manner under particular circumstances.

Secondly, it was shown that the activation of the ß2-AR may play a role in the formation of amyloid β (Aβ). ß2-AR agonists activate the gamma-secretase, forming Aβ from the amyloid precursor proteins (APP), resulting in an elevated accumulation of Aβ [14]. Moreover, a microperfusion of Aβ1–40 in combination with the ß2-adrenergic agonist clenbuterol into the hippocampus caused an augmented degradation of the Aβ1:40 peptide in comparison to the controls without clenbuterol [60]. Some of these degradation products of the long-chain Aβ and Aβ1–40 and Aβ1–42 itself can stimulate different GPCR in a similar manner as the classical receptor agonists. For example, the Aβ-fragments Aβ25–35 and Aβ10–37 activate the α1-AR and this activation was abolished by the antagonist prazosin or urapidil. These Aβ-peptides recognize the first extracellular loop of the α1-adrenergic receptor and the Aβ25–35 and Aβ10–37 induced activation could be blocked by peptides corresponding to the first extracellular loop [61]. Such α1-adrenoceptor antibodies can influence moderately the contractility of the coronary and middle cerebral artery and strongly the renal artery of the rat [62]. Moreover, Tohda et al. demonstrated that Aβ 25–35 induced memory impairment, axonal atrophy and synaptic loss [63].

Wang and coworker observed in prefrontal cortical neurons that Aβ1–40 and Aβ1–42 can bind to the β2-AR, and activate the protein kinase A (PKA) in these [64,65]. This observation was not confirmed in our experiments using a functional cardiomyocyte assay. In our bioassay Aβ 1–40, Aβ1–42 but also Aβ1–14 activate the fast estradiol receptor GPR 30 (unpublished data). In contrast, the N-terminal truncated Aβ peptide Aβ-[Pyr]3–43 that representing a major Aβ plaque component was able to activate the β2-AR subtype in our cardiomyocyte model. This Aβ peptide, activate the ß2-AR in similar manner as the agonist clenbuterol. This activation was neither influenced by the α1-adrenergic antagonist urapidil nor the β1-antagonist bisoprolol. Only the β2-antagonist ICI118.551 was able to block the activity induced by Aβ- [Pyr]3–43. Therefore, we assume that in the experiments of Wang on neuronal tissues the Aβ-peptides Aβ1–40 and Aβ1–42 were truncated by endogenous peptidases to Aβ-[Pyr]3–43 that can than recognize the β2-AR subtype.

A third possibility to influence the β2-AR activity can be seen in fact that the long- chain Aβ peptides Aβ1–42, Aβ1–40, and Aβ10–37, yet not the short breaking products of the amyloid Aβ1–14, Aβ25–35 and Aβ28–40 prevent desensitization of the β2-adrenergic agonist clenbuterol. The combination of the long chain Aβ peptides and clenbuterol induced a permanent activation of the β2-signal cascade similar to the response seen for the agonist-like β2-AAbs. The β2-AAbs induced their long-lasting effect via a cross-link between the two arms of the antibody molecule and two receptors. This cross-link stabilized the active conformation of the receptor and led to a permanent stimulation (as described above). Why the long-chain Aβ-peptides can prevent the desensitization of the adrenoceptors is an open question and under investigation. Yet, we were able to show that other proteins for example the C-reactive protein (CRP) can also prevent the desensitization of different GPCRs [66]. Moreover, the unspecific matrix-metalloprotease (MMP) inhibitor GM6001 prevented the desensitization of the adrenoceptors [67]. Therefore, is it assumable that proteases may play an important role in the process of desensitization. These observations indicate that the long chain Aβ can also contribute to the adrenergic overdrive.

We hypothesize that a dysregulated β2-mediated signal cascade might be a molecular mechanism of an autonomic dysregulation, both observed in neurodegenerative disorders, e.g. glaucoma and AD. A seropositivity for β2-AAb and the β-adrenergic agonists itself can enhance β2-AR activation. In addition to β2-AAb, the Aβ peptide Aβ-[Pyr]3–43 was also able to activate the β2-AR mediated signal cascade directly. Furthermore, the long chain Aβ peptides induced like the β2-AAb a permanent stimulation and deactivate the protection mechanism of the cells against an adrenergic overstimulation. All these mechanisms may play an important role in the pathogenesis of glaucoma and AD.

## Conclusion

Glaucoma and AD are known to be neurodegenerative disorders with an involvement of autoimmune mechanisms and impaired microcirculation. As both disease entities showed a seropositivity for β2-AAb we postulate that an overstimulated ß2-AR pathway can induce an adrenergic overdrive, which can be enhanced by amyloid-β peptides in patients with AD.

## Supporting information

S1 File (DOCX)
