# Supplementary material for: Glaucoma and Alzheimer: Neurodegenerative disorders show an adrenergic dysbalance
Source: PLoS One. 2022 Oct 6;17(10):e0272811. doi: 10.1371/journal.pone.0272811 (PMC9536590; doi:10.1371/journal.pone.0272811)
Supplement: S1 File — (DOCX) [file pone.0272811.s001.docx]

Supplementary:

Data of the figures and further experiments (each point represents the mean value of 10 different measurement points / culture flask).

Regarding Figure 2

| **Clenbuterol** 1µM | | | | |
| --- | --- | --- | --- | --- |
| Clen. |  |  |  | Clen. |
| 5‘ | 60‘ | 120‘ | 125‘ | 130‘ |
| 10.17 | 10.75 | 4.67 | 0.50 | 3.33 |
| 10.00 | 9.0 | 7.17 | 0.17 | 2.83 |
| 13.17 | 9.5 | 6.33 | 0.50 | 2.67 |
| 8.83 |  | 3.67 | -0.50 | 2.33 |
| 10.75 |  | 7.63 | 0.25 | 2.13 |

| **β2-AR AAB** dilution 1:40 | | | | |
| --- | --- | --- | --- | --- |
| AAb |  |  |  | +Clen.1µM |
| 5‘ | 60‘ | 120‘ | 125‘ | 130‘ |
| 2.63 | 6.38 | 6.75 | 1.43 | 11.38 |
| 3.77 | 6.88 | 7.33 | 0.71 | 10.89 |
| 4.0 | 6.38 | 7.0 | -0.13 | 10.25 |
|  |  |  |  |  |
|  |  |  |  |  |

Regarding Figure 3

| **Aβ Pyr 3-43** | | | |
| --- | --- | --- | --- |
| Pyr 3-43 | +Urapidil 1µM | +Bisoprolol 1µM | +ICI118.551 0.1µM |
| 5.83 | 6.00 | 5.17 | 0.00 |
| 5.67 | 6.00 | 5.17 | 0.17 |
| 6.17 |  |  | 0.33 |
| 5.83 |  |  | -0.17 |
| 6.83 |  |  |  |
|  |  |  |  |

| Clenbuterol 1µM + **Aβ 1-14**, 0.1µM | | | | |
| --- | --- | --- | --- | --- |
| Clen. |  |  |  | Clen. |
| 5‘ | 60‘ | 120‘ | 125‘ | 130‘ |
| 7.83 | 5.83 | 3.17 | -0.83 | 2.33 |
| 5.00 | 3.33 | 3.0 | 0.67 | 2.67 |
| 6.67 | 5.83 | 4.83 | 0.17 | 2.33 |
| 11.83 | 8.83 | 2.17 | 0.67 | 2.17 |
| 12.17 | 9.50 | 4.50 | 0.33 | 2.33 |

Regarding Figure 4

| Clenbuterol 1µM + **Aβ 1-40**, 0.1µM | | | | |
| --- | --- | --- | --- | --- |
| Clen. |  |  |  |  |
| 5‘ | 60‘ | 120‘ | 125‘ | 130‘ |
| 10.17 | 10.67 | 10.33 | 0.67 | 10.33 |
| 9.17 | 8.83 | 9.67 | -0.67 | 9.17 |
| 11.50 | 12.67 | 11.50 | 0.33 | 10.17 |
| 11.00 | 10.67 | 10.25 | 0.00 | 9.00 |
| 9.33 |  | 8.86 | -0.25 | 7.25 |
| 9.00 |  | 8.25 |  |  |

Further experiments

| Clenbuterol 1µM + **Aβ 1-42**, 0.1µM | | | | |
| --- | --- | --- | --- | --- |
| Clen. |  |  |  | Clen. |
| 5‘ | 60‘ | 120‘ | 125‘ | 130‘ |
| 9.00 | 9.00 | 7.33 | -0.50 | 10.83 |
| 8.17 | 8.00 | 8.00 | 0.33 | 8.25 |
| 9.50 | 10.83 | 11.33 | 0.17 | 10.17 |
| 8.83 | 9.33 | 8.83 | 0.67 | 9.33 |

| Clenbuterol 1µM + **Aβ 10-37,** 0.1µM | | | | |
| --- | --- | --- | --- | --- |
| Clen. |  |  |  | Clen. |
| 5‘ | 60‘ | 120‘ | 125‘ | 130‘ |
| 10.50 | 11.33 | 11.25 | 0.50 | 11.00 |
| 8.33 | 9.17 | 7.17 | 0.67 | 10.33 |
| 9.33 | 9.83 | 6.00 | 0.00 | 9.50 |

| Clenbuterol 1µM + **Aβ 25-35**, 0.1µM | | | | |
| --- | --- | --- | --- | --- |
| Clen. |  |  |  | Clen. |
| 5‘ | 60‘ | 120‘ | 125‘ | 130‘ |
| 10.67 | 8.33 | 7.33 | 0.17 | 2.83 |
| 8.67 | 9.17 | 4.83 | 0.17 | 3.50 |
| 5.33 | 4.33 | 4.67 | 0.50 | 2.50 |
| 11.00 | 7.83 | 6.00 | 0.00 | 2.83 |

| Clenbuterol 1µM + **Aβ 28-40**, 0.1µM | | | | |
| --- | --- | --- | --- | --- |
| Clen. |  |  |  | Clen. |
| 5‘ | 60‘ | 120‘ | 125‘ | 130‘ |
| 10.50 | 7.83 | 4.67 | -0.50 | 2.50 |
| 9.00 | 7.83 | 4.17 | 0.33 | 4.00 |
